# Risk-Taking Behaviors of Adult Bedridden Patients in Neurosurgery: What Could/Should We Do?

**DOI:** 10.3389/fmed.2021.676538

**Published:** 2021-08-10

**Authors:** Jean-Jacques Lemaire, Rémi Chaix, Anna Sontheimer, Jérôme Coste, Marie-Anne Cousseau, Charlène Dubois, Mélanie San Juan, Christelle Massa, Sandrine Raynaud, Alexandra Usclade, Bénédicte Pontier, Youssef El Ouadih, Kamel Abdelouahab, Luca Maggiani, François Berry

**Affiliations:** ^1^Institut Pascal, Université Clermont Auvergne, CNRS, INP Clermont-Auvergne, Clermont-Ferrand, France; ^2^Service de neurochirurgie, CHU Clermont-Ferrand, Clermont-Ferrand, France; ^3^Direction de la Recherche et de l'innovation, CHU Clermont-Ferrand, Clermont-Ferrand, France; ^4^Sma-RTy SAS, Aubière, France

**Keywords:** risk-taking behaviors, monitoring, bedridden, neurosurgery, risk

## Abstract

Risk-taking behaviors of adult bedridden patients in neurosurgery are frequent, however little analyzed. We aimed to estimate from the literature and our clinical experience the incidence of the different clinical pictures. Risk-taking behaviors seem to be more frequent than reported. They are often minor, but they can lead to death, irrespective of the prescription of physical or chemical constraints. We also aimed to contextualize the risks, and to describe the means reducing the consequences for the patients. Two main conditions were identified, the loss of awareness of risk-taking behaviors by the patient, and uncontrolled body motions. Besides, current experience feedback analyses and new non-exclusive technological solutions could limit the complications, while improving prevention with wearable systems, neighborhood sensors, or room monitoring and service robots. Further research is mandatory to develop efficient and reliable systems avoiding complications and saving lives. Ethical and legal issues must also be accounted for, notably concerning the privacy of patients and caregivers.

## Introduction

Agitated, confused, and also moderately disabled in-hospital patients can injure themselves, even if they are bedridden. Indeed, as soon as a patient cannot manage wide-amplitude movements, purposeful or not, and either during motion or control of body stability, of the head, the trunk, and limbs, there is a risk of injury and even death. In neurosurgery, a large population of patients is potentially concerned because of the large typology of diseases such as traumas, tumors, strokes, neuralgia, and functional disorders. The risks are all the more important and frequent as the population accessing the care increases inevitably as patients get older and are more vulnerable and frail, whatever the causes. As a result, the number of neurological diseases increases regardless of the symptoms at admission. This in turn affects the risk of developing neuropsychological disorders during primary care.

Physical and chemical restrains are proposed for the most confused and agitated patients to limit the consequences of risk-taking behaviors. However, in spite of such therapeutic options, patients can fall off their bed or can die of asphyxia while trapped by the restraint system. Forty deaths over 10 years were reported in 2006 in France (1). This small number of dramatic consequences is likely underestimated as evoked in Canada in 2010 (2). A French insurance company suggested that the number of cases will increase over time (3). Since the recent introduction, in hospitals, of reporting serious adverse events related to care (SAEC) in France (4), 26 out 820 SAEC (extracted from 3,500 primary registrations), thus 3.2%, were related to “poorly controlled” passive physical constraints in psychiatric and elderly persons. As of 2,000, the risk of death due to passive constraints for institutionalized elderly persons was estimated to 1/1,000, of which 42% happened in bedridden people (5). The increasing number of individual rooms enabling better confidentiality and privacy, can also increase the number of falls (6, 7) and the delay before the discovery of the accident**. **The magnitude of the risk, however, should be limited (8). The organization of care by the nurses and doctors directly impacts the consequences of risk-taking behaviors. These behaviors are intrinsically random, occur in *bursts*, all along the nychthemeron, and that, in turns, requires a quasi-permanent screening of the patients. Indeed, practically the risk-taking patients are, most of the time, alone, because no hospital has enough human means to maintain continuous surveillance. Hence the detection of these risk-taking conditions up to death depends on the organization of care 24 h a day. For instance, in neurosurgery a care giver visits a patient in routine every hour during the daytime, but it can be less often at night. Hence without monitoring and alerts, the patient can suffer for at least up to 1 h, with potential severe consequences. The visits of relatives can also be an opportunity to detect risk-taking behaviors or their consequences, as well as the alert of the roommate, if any and with the capacity to do so. One can easily understand that the discovery by relatives, of their relatives exhibiting risk-taking behaviors, or in a life-threatening condition, or worse, either with or without physical or chemical constraints, is taken very badly, whatever the context. Even with interesting advanced programs of the organization of care [see e.g., (9)], there is a real need to efficiently and specifically monitor these patients. In parallel, the *numerous* factors leading to adverse effects to in-hospital patients are better identified, roughly estimated at 10%, and are about surgery, drugs, and infections (10). The specific risk of falls, that covers a large range of conditions, is nowadays well-identified (11). On the contrary, there is little interest in other accidental injuries or adverse effects, particularly of bedridden patients including the use of physical and chemical restraints (12). Recent studies on the global rate of deaths of bedridden patients during current short hospital stays (about 2 weeks) was evaluated at 1.4% in China, but there are no data on in-hospital accidents (13). The global risks linked to bedridden patients are known, they are more prone to pressure ulcers, pneumonia, deep vein thrombosis, and urinary tract infections (14). In poor hospitals around the world, the importance of alertness, gait, and being bedridden are also well identified independent factors on outcomes (15).

During in-hospital stays, risk-taking behaviors that might also result in death are likely rare, but undoubtedly underestimated. Large studies on adverse effects that analyzed incomplete data or with a too coarse-mesh filter thus failed to determine the precise number of accidents and the causes. There are several conceivable reasons. The caregivers are not always inclined to declare accidents, except when they are severe, because of technical issues to prepare the statement, lack of time, and potentially because they could be afraid of the consequences to their employment depending on whether they are responsible or not. The patients are not always able to tell that they had an accident, in particular if there is a temporary or chronic state of confusion. Relatives are not always aware of accidents, except if they are severe or if they were alerted by the medical staff. There is a lack of data on insurance's reimbursement policies on risk-taking behavior consequences. It seems that public health organisms and agencies focused more on frequent and large questions than on such complex issues. Finally, there are also specificities by country with regard to historical, ethical, legal, and societal conditions [see e.g., (16)].

Considering the lack of evidence on risk-taking behaviors, and the true potentially severe consequences of death at the individual level, we aimed to review the different conditions linked to risk-taking behaviors and the different clinical pictures of such behavior in neurosurgery. The goal is to envisage different ways to limit *the likelihood and* the consequences of the risk-taking behaviors of bedridden patients, from restraints techniques to continuous monitoring. Some aspects of medical and organizational means of prevention could be also inferred from the clinical pictures.

## Risk-Taking Behaviors of Bedridden Patients in Neurosurgery

### Global Care and Medical Conditions

Neurosurgical patients are mostly bedridden, at least temporarily because of the frequent severe disability linked to the disease, which is either treated or monitored. Some are more prone to risk-taking behaviors, particularly during preadmission and post-admission in intensive care units and operating rooms, whatever the circumstances, as well as those hospitalized in continuous monitoring facilities depending on neurosurgical units. In parallel, the number of neurosurgical patients, notably those suffering from head injury, who are not hospitalized in a neurosurgical unit is not negligible considering the evidence, but cannot be precisely estimated because of the lack of data. This is the consequence of the structural and/or functional organizations of care, which are different from one hospital to another, one region to another, one country to another, and the moving evolution of medico-economic concepts. The ongoing attempt of economic rationalization of parameters such as beds/medical insurance care costs/disease patients, indirectly gives us some elements that illustrate the complex and hidden problems of the variety of cares and thus of the number of neurosurgical patients across the different hospital facilities (17–19). The way the hospitalization is *organized* in each country is so different across the world that it is not possible to have an overview of the number and severity of risk-taking patients, and the capability to minimize the coming or observed complications. There is also little interest in large detailed data merging of the pathologies, the hospitalization facilities, and the country health policy, except, for example, at a global macroscale in surgery (20). However, medico-economic studies have harvested data showing the wide range of pathologies treated in neurosurgery, the demographic evolution (21, 22), and the main causes of claims (23). Limited data are also available in countries with limited neurosurgical facilities (24, 25).

### Clinical Pictures

Risk-taking behaviors of bedridden patients can be split into two main clinical pictures, (i) the loss of awareness of risk-taking behaviors by the patient, and (ii) uncontrolled body motions. The loss of awareness of risk-taking behaviors is typically observed with confused patients or patients suffering from disorders of consciousness. Loss of consciousness is a common feature, at least partially, of the self and the environment, without coma, that leads to a confusion fluctuating state with a dream-like experience. The anosognosia, or the loss of awareness of deficit, can be associated with confusion. Hence the clinical pictures can be very different and complex, all the more, when the patients are agitated. This condition of loss of awareness of risk-behaviors is caused or worsened by a lot of acquired and preexisting pathologies, such as pain, mood fluctuations, biologic disorders, neuropsychiatric disease, or sequelae of head injury. Uncontrolled body motions can also lead to risk-taking behaviors. Of course, this is always the case for loss of awareness risk-taking behaviors, but also for conscious patients who move their body, head, trunk, and limbs where the motion is not controlled, whether by clumsiness, or because of restraining systems or any kind of obstacle (perfusion, orthopedic systems, pillows…), or due to neuro-orthopedic troubles. In neurosurgery, a particular condition is the cranial traction of a patient with instable cervical fractures, where hazardous mobilization, whatever the cause, can be dramatic.

## What Could/Should We Do?

There are different ways to anticipate and to monitor risk-taking behaviors of bedridden patients, in general, and notably in neurosurgery. The challenge is ambitious and should include all the actors from the ground to the decision-makers and biomedical companies.

The prevention relies on the awareness of such behaviors, and the deep analysis of the conditions that can generate them, as from the level of care teams. There are different ways to optimize the risk in the context of risk-taking behaviors of bedridden patients, for instance, following an International Organization for Standardization risk approach, by reducing the likelihood of falls, reducing the consequences of such falls, losing the minimum of benefits for the well-being of a patient, and preventing the introduction of new risks because of the treatment of the first one. The aim is to minimize and optimize all elements, from those which can be seen as insignificant such as clearing the bed and its surroundings of all unnecessary objects and checking the efficiency of the alarm systems, to more evident parameters such as optimal pain treatments, optimization of sedative drugs and restraints systems, and appropriate organized schedules of visits, either nurses or relatives. These elements can be improved directly in the frame of experience feedback forms (26) and with the help of SAEC. Environmental modifications can also improve surveillance and limit the diversion of patients, such as decentralization of alert systems (portable tools for the nursing staff; see e.g., below) and avoidance of elements that lead to interpretation (e.g., abstract images). However, whatever the optimization of care organization and mandatory pre-conditioning, at some point, only new technologies can truly impact the incurred risks of each individual patient. Indeed, technical monitoring should be developed because permanent, bedside, manned surveillance is not realistic. Three main tracks, eventually complementary, can be roughly envisaged: wearable systems; neighborhood sensors fixed to objects such as the bed and restraint systems, or room monitoring; and service robots. Wearable systems have been developed, such as a waist-mounted, triaxial accelerometer (27) and inertial sensor units (28). Sensors fixed to the bed have been proposed such as load cells (29). Direct video monitoring is attractive but remains difficult as the privacy of patients can be violated. In France, patients cannot be filmed or photographed in their hospital bedroom, unless they expressly authorize it or if they film or photograph themselves. The French Restatement of article 9 of the French Code Civil applies for this subject: “*Everyone has the right to respect for his private life*.” However, the patient can sign an authorization allowing the hospital to film or take a picture for a purpose that must be determined. Indeed it should be noted that to be operational and enforceable, the authorization must define the terms of dissemination of the image with regard to duration, medium, and geographic area. For research and future medical monitoring aiming to protect patients, such as those bedridden with risk-taking behaviors, filming should become legal provided that the patient or the representative has been able to consent to the use of a device. The images should of course not be used for any purpose other than prevention of risks and will therefore only be used by the healthcare and medical team in charge of the patient in strict compliance with the General Data Protection Regulation (30). Nevertheless, room monitoring systems that use video monitoring have already been successfully proposed, notably to reduce the number of falls while reducing sitter usage (31, 32). To date, there are no service robots dedicated to monitoring risk-taking behaviors of bedridden patients. Such a robot, i.e., that performs useful tasks for humans or equipment excluding industrial automation application, could potentially have functionalities dedicated to the monitoring of behavior of bedridden patients, and this potentially in a collaborative manner with room monitoring systems. Beyond the monitoring of behaviors, the integration of the data or processed data into the care workflow is not yet fixed, but recent advances in machine learning suggest that it could be done in the near future (33–35). There is a growing interest in big data, including on nurses (36), to improve care. Whatever the solutions proposed, usability will remain a key point (37).

## Conclusion

There is no doubt that the monitoring of risk-taking behaviors of bedridden patients will emerge in the very near future, enabling health care providers to save lives and avoid severe complications. In the meantime, besides the technical issues, ethical and legal concerns must be solved, according to each country. It is also reasonable to *estimate* that monitoring could also be useful to assess the impact and efficacy of physical and chemical restraints, like for any treatments (4, 38), and beyond such as pressure injury prevention (39). Legal, health, insurance, and ethical safeguards and authorities that can oversee, with care, the developments of these emerging technologies in quality frameworks such as clinical governance (40).

## Author Contributions

J-JL, RC, and FB: conceptualization. J-JL, FB, RC, M-AC, AS, JC, KA, and LM: writing—original draft. CD, MS, CM, SR, AU, BP, and YE: writing—substantial review and corrections. J-JL and FB: supervision. All authors have read and agreed to the submission of the manuscript.

## Conflict of Interest

The authors declare that the research was conducted in the absence of any commercial or financial relationships that could be construed as a potential conflict of interest.

## Publisher's Note

All claims expressed in this article are solely those of the authors and do not necessarily represent those of their affiliated organizations, or those of the publisher, the editors and the reviewers. Any product that may be evaluated in this article, or claim that may be made by its manufacturer, is not guaranteed or endorsed by the publisher.
